# Acute Toxicity of Dental Gel Based on *Origanum vulgare* in Mice

**DOI:** 10.1155/2023/6691694

**Published:** 2023-09-14

**Authors:** Karakoz Badekova, Gayane Atazhanova, Irina Losseva, Aigul Medeshova, Ailazzat Aitkenova, Anar Brazhanova

**Affiliations:** Institute of Life Science, NC JSC “Karaganda Medical University”, Karaganda, Kazakhstan

## Abstract

**Objectives:**

The creation of new herbal medicines for their use in dentistry is relevant. The purpose of this work is to study the acute toxicity of the anticaries dental gel (ACDG3) developed by us based on *Origanum vulgare*.

**Results:**

In case of studying the safety of anticaries dental gel “ACDG3” in animals, that with a single dose of up to 2000 mg/kg, the absence of pathological changes in the behavior of animals was noted. Biochemical studies indicate that the studied doses of dental gel did not lead to significant deviations in the blood parameters of mice and deviations fluctuated within the reference values. According to the results of a morphometric study conducted 15 days after the administration of the drug, no deviations were found. The histological evaluation of organs showed little change in the cardiac architecture in animals treated with ACDG3 at doses of 1000 mg/kg and 2000 mg/kg. On the other hand, no significant changes in the cardiac function were observed in all treated mice.

**Conclusion:**

As follows from the results obtained, in case of determining acute toxicity, the studied anticaries gel, ACDG3, showed low toxicity. For mice, LD50 was 2000 mg/kg intragastrically. So, according to the generally accepted classification of the toxicity of substances, ACDG3 can be attributed to the class of low-toxic substances (IV class of toxicity, LD50 > 5000 mg/kg, intragastric administration), that is, to practically nontoxic compounds.

## 1. Introduction

Herbal medicines and preparations containing herbal components have been successfully used in dentistry for a long time [1–10]. Despite this, interest in drugs based on medicinal plant materials is increasing every year. This trend of reviving interest in medicinal plants is explained by the following disadvantages of synthetic drugs: toxicity, side effects, and allergies [11].

*Origanum vulgare* has been traditionally used for centuries to flavor foods and treat various diseases due to its high essential oil content [12]. The long-standing use of *Origanum vulgare* in folk medicine is generating even more interest in the design of new pharmaceuticals. New studies have shown medicinal properties such as antimicrobial [13], antiviral, antioxidant [14], anti-inflammatory, antispasmodic [15], antiurolytic [16], antiproliferative [13], and neuroprotective [17].

Before submitting a new pharmacological agent of any origin for clinical study, it is necessary to make sure that it will not harm a person. The preclinical study of the toxicity of new drugs ensures the relative safety of clinical trials for volunteers or patients [18, 19]. In recent years, the problem of drug safety has become one of the most pressing health problems in the world. An important indicator of drug safety is acute toxicity [20].

The article presents the results of studying the acute toxicity of the anticaries dental gel (ACDG3) developed by us based on *Origanum vulgare* [21].

## 2. Methods

Acute toxicity studies were performed on white outbred mice, males and females weighing 23–36 g. All animals were randomized into four groups of five animals each. The selection was performed by the random sampling method considering the body weight. The animals were acclimatized in the conditions of the test room for 14 days. The animals were kept under standard vivarium conditions on a normal diet with free access to water and food.

All manipulations with animals were performed according to the rules adopted by the European Convention for the Protection of Vertebrate Animals used for research and other scientific purposes [22].

To study acute toxicity (dosage form: gel), the drug “ACDG3” was administered to experimental animals intragastrically using a special probe. Animals received a physiologically acceptable amount of the drug once. For the study of acute toxicity, doses of 500 mg/kg, 1000 mg/kg, and 2000 mg/kg were selected as recommended for the study. Each dose of the compound was administered to experimental animals divided into 4 groups (5 mice of both sexes) based on the body weight. Group A (males) received the gel at a dosage of 500 mg/kg, group B (males) received the gel at a dosage of 1000 mg/kg, group C (females) received the gel at a dosage of 2000 mg/kg, and group D (control, females) received distilled water in a volume of 1 ml. The calculated dose required for administration was dissolved in 0.2 ml of water. Animals were weighed during the formation of groups, before the introduction of the test gel, as well as on days 1, 5, 10, and 14 after the administration of the gel. Before and after the administration of the substance, the animals did not receive food for 2-3 hours. The animals were observed for 14 days. The study design is presented in Table 1.

Blood sampling for clinical analysis was performed from the tail vein after 18 hours of fasting. Blood was taken into test tubes with EDTA anticoagulant. Analyses were performed on a Mindray BC-3200 hematology analyzer (Mindray, China).

On the 15^th^ day of the experiment, all animals were taken out of the experiment under anesthesia with further macroscopic and morphometric examination of the organs. After the registration of death, mice were subjected to complete necropsy, which included examination of the external surface of the body, all passages, and the cranial, thoracic, abdominal cavities with organs and tissues.

The lungs, heart, liver, kidneys, spleen, testes, and ovaries were weighed.

### 2.1. Data Processing

All experimental data were processed by the method of variation statistics (accepted significance level:*p* ≤ 0.05). The significance of the results was assessed using Student's *t*-test.

The total duration of observation of animals in the study of acute toxicity is 14 days. On the first day after the introduction of the sample, the animals were under continuous observation. The general condition of the animals, the peculiarities of their behavior, the intensity and nature of motor activity, the dynamics of body weight, and changes in the mass of internal organs were regularly recorded. The degree of toxicity of the drug was assessed by changes in the general condition of animals, mortality, and the effect on the dynamics of body weight of animals.

In case of studying acute toxicity against the background of the introduction of ACDG3, the manifestation and severity of pathological signs were noted as follows: the presence of abnormal postures, abnormal movements, self-injury, tremor, convulsions, and Straube syndrome.

## 3. Results and Discussion

When studying the safety of anticaries gel “ACDG3” in animals, that with a single dose of up to 2000 mg/kg, the absence of pathological changes in the behavior of animals was noted. The general condition and behavior of the animals were normal and did not differ from those in the animals of the control group. The motor activity, reactions to tactile, pain, sound, and light stimuli were unchanged. The intensity and nature of motor activity, coordination of movements, and skeletal muscle tone remained at the same level. Behavioral reactions did not deviate from the standard type. No pathological changes were recorded in the hair, skin and the color of mucosa. During the study period, no statistically, significant change was found in body weight of mice injected with dental gel compared to the indicators of control animals.

The results of the study of acute toxicity with intragastric administration of the tested gel samples are presented in Table 2. There are no significant body weight changes between the groups, which suggest the safety of the anticaries dental gel “ACDG3” at a single dose of up to 2000 mg/kg.

The degree of toxicity of the drug was assessed by changes in the general condition of animals, mortality, and the effect on the dynamics of the body weight of animals. During the study period, the death of animals was not observed (Table 3).

Biochemical studies (Table 4) indicate that the studied doses of dental gel did not lead to significant deviations in the blood parameters of mice and deviations fluctuated within the reference values. The rest of the studied parameters of the clinical analysis of the experimental groups did not differ significantly from the control groups.

According to the results of a morphometric study conducted 15 days after the administration of the drug, no deviations were found. All macroscopically examined organs (the lungs, heart, liver, kidneys, spleen, etc.) did not differ from the control group. The relative mass of the internal organs of mice remained within the physiological norm (Table 5). Table 5 shows that no significant differences were found in the mass ratios of internal organs of experimental animals, which testifies the absence of the toxic effect of the drug on internal organs and systems. Nevertheless, an insignificant increase in the mass of the liver and lungs was observed. In the course of macroscopic examination of internal organs, no differences were found between the experimental and reference groups. When examining the thoracic and abdominal cavities, no disturbances in the location of internal organs were observed. The stomach had the regular shape and size, and the lumen was filled with dense food content. The mucosa of the stomach body was pale pink, shiny, and folded. The size and shape of the liver did not present significant changes. The liver tissue had brownish color and moderately dense consistency. The size and shape of the kidneys did not differ from the reference ones, and the capsule was easily removable. The surface of the organ was smooth and of homogeneous brownish-greyish color.

**Figure 1:**
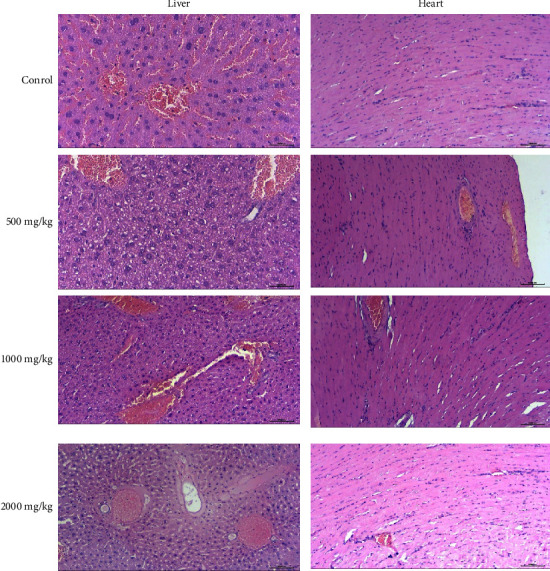
Histology of the liver and heart of mice treated with ACDG3.

The histology of liver sections of control mice showed normal hepatocellular architecture along with well-preserved liver cells, visible central veins, and no histological abnormalities (Figure 1). In animals treated with a dose of 500 mg/kg, no adverse effects on the histoarchitecture of hepatocytes were found. Liver sections taken from the 1000 mg/kg dose group showed some histological changes such as mild sinusoidal dilatation, mild hepatic cord disorganization, steatosis, and hepatocytes. In addition, the administration of ACDG3 at the highest dose (2000 mg/kg) caused a slight change in the histoarchitecture of treated mice, showing inconspicuous central vein congestion, mild hepatic cord disorganization, and binuclear hepatocytes.

The histological evaluation of the heart showed little change in the cardiac architecture in animals treated with ACDG3 at doses of 1000 and 2000 mg/kg. On the other hand, no significant changes in the cardiac function were observed in all treated mice.

In the group of animals treated with ACDG3 at a dose of 1000 mg/kg, normal, well-defined histological structures were observed without signs of vascular changes. Changes in histoarchitecture, portal triads, hepatocytes, sinusoids, and inflammation and the presence of dystrophy, necrosis, and fatty changes in animals in the group in the studied histological sections were not detected.

In addition, in no case was there a change in the diameter and appearance of the central veins, hepatic sinusoids, and portal veins.

During the study period, the animals were neat, active, had a positive appetite, adequately responded to sound and light stimuli, the processes of urination and defecation proceeded normally, and convulsions and respiratory disorders or any other manifestations of toxic effects were not observed. The study of the dynamics of the body weight of animals during the experiment indicates the absence of a toxic effect of the drug on the growth processes of animals.

The study of acute toxicity of the drug during preclinical investigation is an essential step in the drug development process [23]. According to the data of this study, the application of the new dental gel on mice of both genders in doses from 500 to 2000 mg/kg does not cause toxicity.

In the process of analyzing the change in the body weight of mice over 14 days, no significant abrupt fluctuations or decrease in indicators were found compared to the reference group. Body weight growth of all the studied mice proceeded gradually and evenly.

Changes in the hematological characteristics of males and females were detected; differences in parameters such as the number of leukocytes and erythrocytes were observed in the blood of mice as well as small variations were registered in the percentage of lymphocytes, monocytes, and granulocytes. However, these changes in the indicators were not statistically significant (*p* < 0.05) (Table 4).

Small changes in biochemical characteristics of blood serum of both males and females were recorded (Table 4); there is a tendency to increase total protein in all groups, which is quite consistent with the increase in the body weight of both experimental and reference animals.

Examination of the data on organ weight indices (Table 5) did not reveal statistically significant differences between the groups of animals. The body weight and weight parameters of internal organs were within the normal range, which indicated the absence of toxic effect of the drug on the state of internal organs. This fact is also confirmed by the results of the histological study of internal organs conducted on animals receiving the maximum dose of the drug.

Consequently, in accordance with the conclusions of the study of acute toxicity of the new dental gel, its administration to mice of both genders in doses from 500 to 2000 mg/kg has no adverse effect on hematological characteristics of blood, biochemical parameters of blood serum, and organ weight.

This investigation work was carried out at a high level, which is confirmed by the data of the modern scientific literature describing similar studies [3, 18–20, 22, 23]. The obtained findings have an important significance as they confirm the toxicological safety of the dental gel and allow us to recommend it for further clinical trials as a tool of dental therapy.

## 4. Conclusion

As follows from the results obtained, when determining acute toxicity, the studied anticaries gel, ACDG3, showed low toxicity. For mice, LD50 was 2000 mg/kg intragastrically. So, according to the generally accepted classification of the toxicity of substances, ACDG3 can be attributed to the class of low-toxic substances (IV class of toxicity, LD50 > 5000 mg/kg, intragastric administration), that is, to practically nontoxic compounds.

## Figures and Tables

**Table 1:** Design of the study of acute toxicity of anticaries gel, ACDG3, when administered intragastrically in mice.

Group nos.	Gender (М/F)	Number of animals	Drug	Dose of drug mg/kg
Mice				
A	M	5	ACDG3	500
B	M	5		1000
C	F	5		2000
D (control)	F	5		—

**Table 2:** Change in the body weight of animals after a single injection of ACDG3.

Doses (mg/kg)	Average weight of animals (g)				
	Initial mass	1 day	5 day	10 day	14 day
Mice					
500	28.5 ± 1.4	28.1 ± 1.2	29.4 ± 1.3	30.9 ± 1.6	31.2 ± 2.5
1000	29.5 ± 3.06	29.3 ± 2.57	30.5 ± 1.5	31.6 ± 2.36	32.72 ± 2.6
2000	32.7 ± 1.52	32.2 ± 1.56	33.5 ± 2.7	35.72 ± 1.51	36.96 ± 1.03
Control	30.8 ± 3.27	30.5 ± 3.42	31.4 ± 2.53	32.65 ± 1.34	33.62 ± 1.43

*Note*. *p* < 0.05 compared to the values in animals of the control group.

**Table 3:** Lethal effects (fall/total) with intragastric administration of anticaries dental gel “ACDG3” in mice.

Dose (mg/kg)	500	1000	20000	Control
Mice	0/5	0/5	0/5	0/5

**Table 4:** Mean blood tests of mice.

Mice	Parameters	Doses (mg/kg)			
		500	1000	20000	Control
Males	WBC (10^9^L)	4.1 ± 0.21	5.3 ± 0.11	5.2 ± 0.9	5.3 ± 0.28
	RBC (10^12^L)	8.8 ± 1.1	8.6 ± 0.2	8.7 ± 1.6	8.8 ± 1.22
	HGB (g/dL)	13.6 ± 0.6	13.9 ± 0.13	13.5 ± 0.3	13.9 ± 0.7
	HCT (%)	36.2 ± 1.16	34.1 ± 1.51	33.04 ± 0.04	36.2 ± 0.2
	MCV (fl)	47.1 ± 0.22	46.9 ± 0.45	48.4 ± 1.3	47.6 ± 2.1
	MCH (pg)	16.2 ± 1.13	17.63 ± 1.09	16.58 ± 0.5	17.75 ± 0.2
	MCHC (g/dL)	35.5 ± 0.32	34.2 ± 0.19	33.2 ± 1.56	35.5 ± 0.7
	PLT (10^9^L)	759 ± 42.2	755 ± 17.3	749 ± 76.2	756 ± 45.1
	PCT (%)	1.5 ± 1.16	1.7 ± 0.8	1.9 ± 0.22	1.7 ± 0.3

Females	WBC (10^9^L)	4.8 ± 0.1	4.3 ± 0.9	5.2 ± 0.2	5.1 ± 0.7
	RBC (10^12^L)	9.7 ± 1.3	9.3 ± 2.6	9, 5 ± 1.2	9.2 ± 1.24
	HGB (g/dL)	14.8 ± 0.82	15.6 ± 0.44	12.5 ± 0.19	12.2 ± 0.7
	HCT (%)	39.2 ± 1.54	41.1 ± 1.7	44.04 ± 0.21	42.2 ± 0.5
	MCV (fl)	47.1 ± 0.11	45.9 ± 0.56	46.4 ± 2.3	49.6 ± 4.8
	MCH (pg)	16.13 ± 1.23	16.3 ± 1.19	16.42 ± 2.2	16.25 ± 0.18
	MCHC (g/dL)	36.5 ± 0.35	36.2 ± 0.11	36.2 ± 1.43	35.5 ± 0.22
	PLT (10^9^L)	765 ± 22.3	788 ± 28.3	745 ± 31.0	740 ± 29.2
	PCT (%)	1.2 ± 0.21	1.2 ± 0.25	1.5 ± 0.55	1.5 ± 0.11

**Table 5:** Average weight of the internal organs of mice.

Mice	Organs	Relative weight of the organ			
		500 mg/kg	1000 mg/kg	2000 mg/kg	Control
Males	Lungs	0.62 ± 0.02	0.60 ± 0.03	0.65 ± 0.4	0.63 ± 0.08
	Heart	0.42 ± 0.46	0.45 ± 0.28	0.43 ± 0.06	0.44 ± 0.03
	Liver	1.5 ± 0.09	1.6 ± 0.04	1.4 ± 0.02	1.5 ± 0.7
	Kidneys	0.5 ± 0.5	0.7 ± 0.6	0.5 ± 0.42	0.5 ± 0.09
	Spleen	0.3 ± 0.65	0.3 ± 0.09	0.2 ± 0.28	0.3 ± 0.26
	Testis	0.1 ± 0.7	0.2 ± 0.76	0.15 ± 0.8	0.2 ± 0.10

Females	Lungs	0.47 ± 0.32	0.45 ± 0.07	0.43 ± 0.45	0.44 ± 0.26
	Heart	0.37 ± 0.06	0.32 ± 0.02	0.32 ± 0.6	0.36 ± 0.2
	Liver	1.2 ± 0.18	1.3 ± 0.12	1.2 ± 0.03	1.3 ± 0.4
	Kidneys	0.3 ± 0.4	0.2 ± 0.4	0.3 ± 0.11	0.2 ± 0.5
	Spleen	0.1 ± 0.5	0.1 ± 0.2	0.1 ± 0.12	0.1 ± 0.4
	Ovary	0.1 ± 0.04	0.1 ± 0.60	0.2 ± 0.9	0.1 ± 0.08

## Data Availability

The data used to support the findings of this study are available at DOI: 10.1016/j.jtumed.2021.05.009.
